# Insights into mammalian biology from the wild house mouse *Mus musculus*

**DOI:** 10.7554/eLife.05959

**Published:** 2015-04-15

**Authors:** Megan Phifer-Rixey, Michael W Nachman

**Affiliations:** Department of Integrative Biology, University of California, Berkeley, Berkeley, United States and Museum of Vertebrate Zoology, University of California, Berkeley, Berkeley, United States; Department of Integrative Biology, University of California, Berkeley, Berkeley, United States and Museum of Vertebrate Zoology, University of California, Berkeley, Berkeley, United States

**Keywords:** the natural history of model organisms, *Mus musculus*, house mouse, evolution, wild mice, classical inbred strain, mouse

## Abstract

The house mouse, *Mus musculus*, was established in the early 1900s as one of the first genetic model organisms owing to its short generation time, comparatively large litters, ease of husbandry, and visible phenotypic variants. For these reasons and because they are mammals, house mice are well suited to serve as models for human phenotypes and disease. House mice in the wild consist of at least three distinct subspecies and harbor extensive genetic and phenotypic variation both within and between these subspecies. Wild mice have been used to study a wide range of biological processes, including immunity, cancer, male sterility, adaptive evolution, and non-Mendelian inheritance. Despite the extensive variation that exists among wild mice, classical laboratory strains are derived from a limited set of founders and thus contain only a small subset of this variation. Continued efforts to study wild house mice and to create new inbred strains from wild populations have the potential to strengthen house mice as a model system.

**DOI:** http://dx.doi.org/10.7554/eLife.05959.001

## Introduction

Today, house mice (*Mus musculus*) are widely known as an excellent mammalian model for studying a wide variety of traits and diseases, including those involved in metabolism, development, neurological disorders, immunity, and others (Morse, 2007). At the beginning of the 20th century, however, Mendel's work had only recently been rediscovered and the race was on to develop genetic model systems. In a series of papers from 1902–1905, Lucien Cuénot used mice to demonstrate Mendel's laws for the first time in mammals (Hickman and Cairns, 2003). Around the same time, William E Castle, a pioneer of the use of *Drosophila* to study genetics, also began investigating the inheritance of coat color in mice (Castle and Allen, 1903). The Castle lab and others launched research programs focused on mouse genetics, and soon realised the need to create inbred strains (see Box 1 for a glossary of specialist terms used in this article) of mice (e.g., Castle and Allen, 1903; Castle and Little, 1910; Russell, 1978; Morse, 1981, 1985). By 1909, the first inbred strain, DBA, was created and the era of modern mouse genetics had begun (Russell, 1978). Since then, hundreds of inbred strains have been developed.

10.7554/eLife.05959.002

Box 1. **Glossary.**

**Adaptive introgression** and adaptive introgressive hybridization****—See ‘Introgression’.

**Association mapping**—A technique in which genetic variation is surveyed to identify statistical associations with phenotypic variation.

**Endogenous retroviral sequences**—Sequences within a genome that are similar to and derived from retroviruses.

**Human commensals**—Organisms that live in close association with humans. The term commonly describes symbiotic relationships where one partner benefits and the other is unaffected. House mice are called commensals despite their potential negative effects on humans.

**Inbred strains**—A strain created by many generations of brother-sister or parent-offspring mating. Historically, strains were considered inbred after 20 such generations, when residual heterozygosity would be vanishingly small. High-throughput genotyping can now evaluate homozygosity as strains are inbred.

**Inbreeding coefficient**—A statistic that summarizes the probability that any two genes in a population are identical by descent.

**Introgression**—The transmission of an allele from one population to another via hybridization and subsequent backcrossing. Adaptive introgression and adaptive introgressive hybridization refer to when an allele is transmitted from one population to another and confers a selective advantage.

**Meiotic drive**—Also called transmission ratio distortion, where two alleles at a locus carried by a heterozygote are transmitted to a zygote unequally (i.e., non-Mendelian inheritance).

**Responder locus**—An allele at a locus that rescues the fertility of sperm carrying a t-haplotype but that does not rescue fertility in wild-type sperm.

**Retrovirus**—A virus that is RNA based and that uses reverse transcriptase to transcribe itself into DNA.

**Robertsonian translocation**—also called Robertsonian fusion, this is a chromosomal rearrangement caused by the fusion of two acrocentric chromosomes into a single metacentric chromosome.

**t-haplotypes**—also called T-alleles, these are variants of a tightly-linked block of genes on the proximal end of chromosome 17 in mice that exhibit meotic drive.

**DOI:** http://dx.doi.org/10.7554/eLife.05959.002

The success of the house mouse as a genetic model organism is largely due to its unique natural history. In this article, we briefly introduce the evolutionary and natural history of house mice, as well as the origins of laboratory strains (Box 2). We highlight several examples in which wild house mice have provided important insights into mammalian biology and suggest future avenues for research.

10.7554/eLife.05959.003

Box 2. **The origins of laboratory mouse strains.**

The trade of mice with distinct coat colors and behaviors has ancient origins in China, Japan, and Europe (reviewed in Keeler, 1931). Mice were used in experimental study as early as the 17th and 18th centuries (Morse, 1981). In the 19th century, Mendel is believed to have worked with mice before switching to pea plants after reportedly being admonished by his bishop for keeping organisms that had sex (Henig, 2000). When Mendel's laws were rediscovered in 1900, researchers saw the advantages of working with a mammal that could be housed in a small area, bred quickly, and that displayed many easily scored, variable traits.

Most of the inbred lines available today have their origins in the trade of fancy mice in the early 1900's (Morse, 1981). Abbie Lathrop, a retired schoolteacher in Granby, Massachusetts, USA, started breeding mice as pets, using mice purchased from other fanciers, including varieties like waltzing mice and creamy buffs. Among her best customers were scientists, including William E Castle and Clarence C Little at Harvard University, and Leo Loeb, who collaborated with Lathrop to investigate mammary tumors in her mouse strains (Morse, 1981). The need for truly genetically homogenous mice spurred these scientists and others to begin inbreeding their colonies. In 1929, CC Little founded the Roscoe B Jackson Memorial Laboratory, which now has the largest selection of inbred mice in the USA. Other major suppliers include Charles River Laboratories, Harlan Laboratories and Taconic Farms, Inc., among others.

**DOI:** http://dx.doi.org/10.7554/eLife.05959.003

## The natural history of house mice

### Phylogenetic history

**Figure 1.**
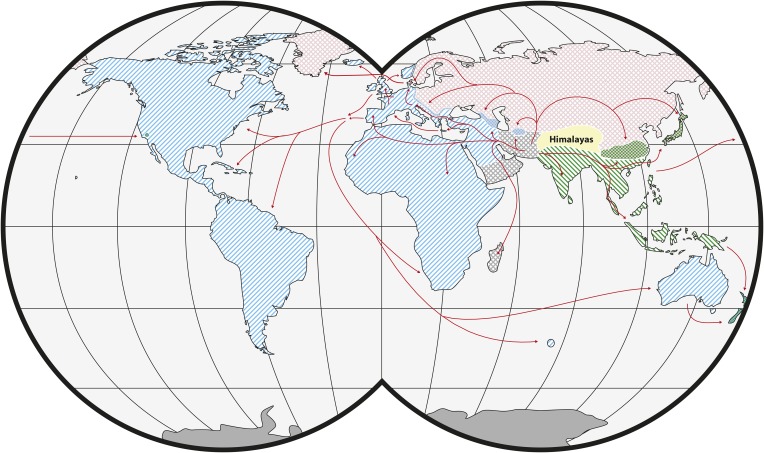
Worldwide distribution of *Mus musculus* subspecies (adapted from Didion and de Villena, 2013). Ranges of *M. musculus* subspecies are indicated by hatching. Green: *M. m. castaneus*; blue: *M. m. domesticus*; red: *M. m. musculus*; grey: central populations and *M. m. gentilulus*. Note that house mice may not be found throughout the complete extent of hatched areas: for example, sub-arctic regions, the Sahara Desert, and the Amazon rainforest. Checkered areas indicate regions of hybridization. Red arrows indicate inferred routes of historical migrations and recent movements in association with humans. Reproduced from Springer and Didion J, de Villena FPM. 2013. Mammalian Genome 24:1–20.

House mice comprise three main subspecies of *M. musculus* with different global distributions: *Mus musculus castaneus*, *Mus musculus domesticus* and *Mus musculus musculus* (Figure 1). Their closest relatives are not human commensals (see ‘Glossary’) and include the mound building mouse, *Mus spicilegus*, and the Algerian mouse, *Mus spretus* (Chevret et al., 2005; Tucker et al., 2005). The ancestral range for *M. musculus* was likely in present-day India (Boursot et al., 1993). Genetic and genomic data indicate that the three subspecies of *M. musculus* started to diverge ∼350–500 thousand years ago (KYA) and that the split among the three subspecies occurred within a short period of time (Geraldes et al., 2008, 2011; Duvaux et al., 2011). Nonetheless, the available evidence suggests that *M. m. castaneus* and *M. m. musculus* are more closely related to each other than either is to *M. m. domesticus* (White et al., 2009; Keane et al., 2011). There is evidence of hybridization among the subspecies in zones of secondary contact (Tucker et al., 1992; Boursot et al., 1993; Duvaux et al., 2011). An additional subspecies, *Mus musculus molossinus*, is found in Japan and is derived from hybridization between *M. m. castaneus* and *M. m. musculus* (Yonekawa et al., 1988). Classical inbred strains of house mice are genetic mosaics of the three main subspecies, although they are primarily *M. m. domesticus* in origin (Yang et al., 2011; Didion and de Villena, 2013). Interestingly, contributions from the other two subspecies may be due to early crosses with *M. m. molossinus*, likely as a result of interactions between mouse fanciers in Japan and Western Europe (Yang et al., 2011). A fifth subspecies, *Mus musculus gentilulus*, has been described from collections in the southern Arabian Peninsula (Harrison, 1972; Harrison and Bates, 1991; Prager et al., 1998). Additional subspecies may yet be defined. In particular, populations in Afghanistan, Pakistan and Iran may be genetically distinct (Rajabi-Maham et al., 2012; Hardouin et al., 2015).

### Habitat and life style

Unique among other species in the genus *Mus*, house mice primarily live in close proximity to humans. Although feral populations exist, house mice are commonly found in residential, agricultural, and commercial structures. The rise of agrarian societies is very recent compared to the divergence among house mice subspecies, indicating that commensalism evolved independently in each of the three subspecies. House mice are omnivorous with varied diets including grains, seeds, and insects (Sage, 1981; Singleton and Krebs, 2007). They generally weigh less than 20 grams and serve as food for predatory snakes, birds and mammals. Laboratory mice tolerate regular handling by humans and live and breed in a small area. Their commensal life style, small size, and flexible diet have likely been key to their success as a model system.

### Reproduction and social structure

The reproductive biology of mice is also favorable for laboratory breeding. Mice have short generation times; gestation lasts approximately 3 weeks and they are sexually mature at 6–8 weeks of age. Thus, generation times in the lab are between 9 and 11 weeks. In the wild, females may breed seasonally with one or two litters per year or they may breed year round if resources are available (Pocock et al., 2004; reviewed in Latham and Mason, 2004; Singleton and Krebs, 2007). In the lab, with unlimited food and good environmental conditions, females can breed year round. Litter sizes in the wild are large (∼4–9 pups), a trait that facilitates the efficient generation of inbred strains (see Sage, 1981; Singleton and Krebs, 2007). House mice in the wild have variable social structures, ranging from discrete ‘demes’ with a single male and one to several adult females and juveniles, to high-density aggregations in which adults are socially gregarious and have largely overlapping ranges (Singleton and Krebs, 2007). While males can be territorial, females are known to mate with multiple males (Dean et al., 2006; Firman and Simmons, 2008). Female house mice tend to live in extended family groups and can nest communally and nurse offspring communally (reviewed in König and Lindholm, 2012). Mice found in a limited geographic area are likely to be related, and the average inbreeding coefficient (see ‘Glossary’) of wild-caught mice in one study was ∼0.2 (Laurie et al., 2007). This natural level of inbreeding may help to purge recessive deleterious alleles and may have facilitated the creation of inbred strains in the laboratory.

### Disease

Wild mice can carry a variety of diseases, including mouse hepatitis virus, mouse mammary tumor virus and mouse parvovirus. They can also be vectors for human diseases, such as leptospirosis, cryptosporidiosis, salmonellosis, and streptobacillosis (summarized in Singleton and Krebbs, 2007). However, unlike rats, they are not known to be major vectors of the plague or of hemorrhagic fevers like hantavirus. They are commonly parasitized by tapeworms, flukes, fleas and lice (Singleton and Krebbs, 2007). As such, wild mice are routinely quarantined before they enter mouse facilities.

### Phenotypic variation

**Figure 2.**
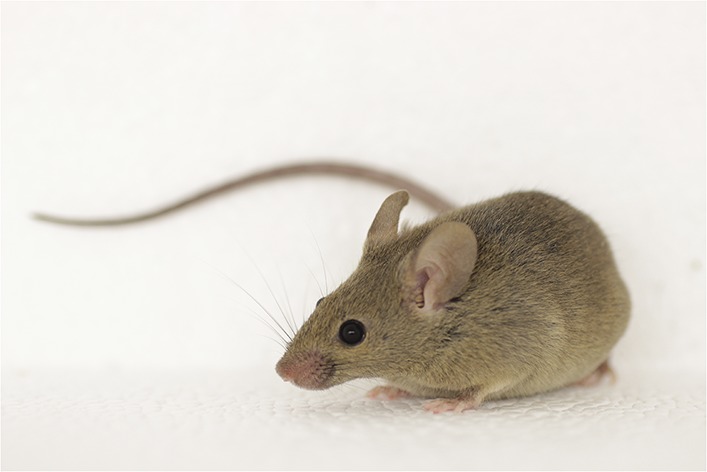
Wild derived house mouse (*Mus musculus domesticus*) from Brazil. Image credit: Taichi Suzuki. **DOI:** http://dx.doi.org/10.7554/eLife.05959.004

Wild house mice (Figure 2) exhibit considerable phenotypic variation, and it is likely that much more remains to be described. In association with humans, house mice have been spread around the world and have adapted to a wide range of environments (e.g., Jones et al., 2012). Mice can be found from sea level to over 4000 m in elevation, from the tropics to subarctic environments, and in both dry and wet environments. Studies of water balance in wild populations suggest that house mice can adapt to arid habitats (Mutze et al., 1991; Moro and Bradshaw, 1999).

Phenotypic variation among wild house mice has been described along environmental gradients. For example, Lynch (1992) surveyed populations of house mice on the east coast of the United States and found that mice from more northern latitudes were bigger and built bigger nests than those from more southern populations. These differences persisted in the lab over several generations, demonstrating a genetic basis. Selection experiments also provide evidence that house mice have the ability to rapidly adapt to cold temperatures. Wild mice bred at 3°C for 10 generations were more fertile, with larger litters of larger body size, and reached sexual maturity earlier than mice bred at 23°C (Barnett and Dickson, 1984). Rapid phenotypic change has occurred on many islands, including shifts in size, morphology, and diet (e.g., Berry et al., 1978; Davis, 1983; Le Roux et al., 2002; Renaud et al., 2013). One example comes from mice on Gough Island, a small island in the Atlantic Ocean approximately halfway between South Africa and Argentina. House mice were likely introduced to this island by sealers or whalers in the 19th century and are the only terrestrial mammals living on it (Holdgate, 1965; Gray et al., 2014). Gough Island mice live independently of humans, are large compared to European house mice, and eat seabird chicks, including those of the critically endangered Tristan albatross (e.g., Rowe–Rowe and Crafford, 1992; Cuthbert and Hilton, 2004; Wanless et al., 2007). These mice are alarming from a conservation perspective, but they are intriguing from an evolutionary one. Variation in natural populations is fertile ground for exploring the genetic basis of complex traits. Such efforts could add to the decades of study of complex traits in inbred strains of mice (such as body size, Cheverud, 2005; Kenney-Hunt et al., 2006).

### Genetic variation

Populations of wild house mice exhibit levels of genetic variation that are several-fold higher than those seen in human populations, consistent with the larger effective population size for house mice (Geraldes et al., 2008, 2011; Halligan et al., 2010, 2013). Patterns of genetic variation in natural populations have provided insight into the demographic and evolutionary history of wild mice (e.g., Bonhomme et al., 2007; Geraldes et al., 2008; Duvaux et al., 2011; Jones et al., 2012) and have helped us to understand how natural selection has shaped patterns of genetic variation (e.g., Halligan et al., 2010, 2013; Phifer-Rixey et al., 2012; for review, Teschke et al., 2012). For example, some beneficial variants appear to have come from introgression between subspecies or between closely related species (Song et al., 2011; Staubach et al., 2012; Liu et al., 2015).

Despite the substantial level of genetic variation seen in wild house mice, the commonly used classical inbred strains of laboratory mice derive from a limited set of founders and thus contain only a small subset of the genetic variation that is seen in nature (Salcedo et al., 2007; Yang et al., 2011). As a result, there are many genomic ‘blind spots’ in crosses involving classical inbred strains. Since the 1970's, new inbred lines have been established using wild mice (Guénet and Bonhomme, 2003). Levels of diversity are much higher among these wild-derived inbred strains than among classical inbred strains (e.g., Ideraabdullah et al., 2004; Yang et al., 2011). Several recent outcrossed populations have also been developed from classical and wild-derived inbred strains, including the Heterogeneous Stock mice (Valdar et al., 2006) and the Collaborative Cross mice (Churchill et al., 2004). Both of these outbred mapping populations contain more genetic variation than do mice derived from crossing classical inbred lines, and they also have more recombination, permitting traits of interest to be mapped with improved resolution (Yalcin et al., 2010). However, they are not without their limitations. First, much of the genetic variation is due to differences between subspecies. Second, since crosses between subspecies result in partial hybrid sterility, selection effectively eliminates genetic variation in some regions of the genome in these crosses. Third, mapping intervals are not typically at the resolution of individual genes. The development of additional inbred lines of wild-derived mice should help address these limitations and augment existing resources.

## Insights from wild house mice

Here, we briefly highlight a few examples in which studies of wild house mice have provided insight into fundamental questions concerning mammalian biology. This is not a comprehensive review; research on wild house mice encompasses a diverse range of topics, only some of which are touched on here. For more information, we refer readers to ‘*The mouse in biomedical research*’ (Foster et al., 1981; Fox et al., 2007), ‘*The genus Mus as a model for evolutionary studies*’ (Britton-Davidian and Searle, 2005) and ‘*Evolution of the house mouse*’ (Macholán et al., 2012).

### Immunity: the Major Histocompatibility Complex

The Major Histocompatibility Complex (MHC) is a family of strikingly polymorphic genes that have a critically important role in the immune response of vertebrates. Proteins encoded by MHC genes bind peptides derived from pathogens and present them on the cell surface for recognition by T cells. MHC genes were first discovered in classical inbred strains of house mice (reviewed in Klein, 1981, 1986; Penn and Musolf, 2012). Wild house mice have also played a major role in advancing our understanding of the MHC. For example, the high level of variation seen at MHC genes was first discovered in populations of wild mice (Klein, 1970; Klein and Bailey, 1971) and has since been documented in many other vertebrates. This discovery spawned a considerable amount of research aimed at understanding the evolutionary forces that maintain such diversity. These studies have led to some intriguing findings regarding the possible functional significance of MHC diversity in wild mice and other organisms. One area of particular interest is mate choice. Data suggest that individual mice prefer mates with dissimilar MHC haplotypes, a form of disassortative mating (e.g., Penn and Potts, 1998; reviewed in Penn and Musolf, 2012). Such mating preferences could function to improve offspring resistance to pathogens, reduce inbreeding, or both. However, the relative importance of MHC in mate choice in wild populations remains unresolved; there are many other factors that have been shown to affect mate choice. Moreover, the specific mechanism by which individuals identify MHC-dissimilar individuals remains obscure.

### Cancer: retroviruses and the Lake Casitas mice

In the late 1960's, virally induced cancers were known to occur in laboratory mice and in chickens, but had yet to be discovered in humans. Robert Huebner and Murray Gardner hypothesized that resistance to viral cancer was likely present in wild mouse populations but had been lost in laboratory strains through inbreeding (Gardner et al., 1991; O'Brien, 2003). Gardner embarked on a mission to sample wild populations of house mice. While he found disease-free mice at most sites, at a poultry farm near Lake Casitas in California, he found ∼85% of the mice carried murine leukemia virus (MuLV). Infected mice developed severe lymphoma and/or hind limb paralysis after sexual maturity (Gardner et al., 1991). Despite the severity of the disease and evidence of transmission, the population did not crash and many mice were apparently unaffected (Gardner et al., 1980, 1991; O'Brien, 2003). Gardener and his colleague, Stephen O'Brien, hypothesized that an anti-retroviral genetic element was segregating in the Lake Casitas mice, protecting some from infection. This proved to be the case; they found an allele at a single locus that conferred complete resistance in homozygotes and was inherited in a Mendelian fashion (Gardner et al., 1980). Researchers in Japan independently identified a common viral cancer, as well as endogenous retroviral sequences (see ‘Glossary’) in *M. m. molossinus* (Ikeda et al., 1981; Odaka et al., 1981). Ultimately, it was shown that resistance in the two populations localized to the same gene (O'Brien et al., 1983).

How did the resistance allele prevent infection? And why were two mouse populations on either side of the Pacific Ocean, thought to be different subspecies, polymorphic for the same protective allele? The resistance locus was itself a truncated endogenous retrovirus in which only the subunit responsible for the creation of the viral envelope remained intact (see ‘Glossary’). When a virus replicates in a cell, some of its envelope proteins bind to receptors on the cell, preventing additional viruses from entering. Thus, resistance to MuLV in the Lake Casitas and Japanese mice was conferred by cells that produce harmless endogenous viral envelope proteins, blocking the binding of the virulent MuLV viruses (Kozak et al., 1984; Ikeda et al., 1985; Dandekar et al., 1987; Ikeda and Sugimura, 1989). These populations of mice share the resistance allele because they have a shared evolutionary history. Japanese mice descend from hybridization between *M. m. castaneus* from Southern China and *M. m. musculus* from northern China. While the resistance gene and the virus were undetected in *M. m. musculus*, they were both found in *M. m. castaneus* (O'Brien, 2003). Although mice in North America were originally believed to be entirely of *M. m. domesticus* origin, the Lake Casitas mice are hybrids of *M. m. domesticus* and *M. m. castaneus* (Orth et al., 1998). The Lake Casitas farm was originally settled by immigrants from China in the 19th century, who likely brought *M. m. castaneus* with them (Gardner et al., 1991). The Lake Casitas mice have provided insight into the mechanisms of resistance to retroviruses, the dynamics of disease outbreaks, and the origins of sequences derived from retroviruses found throughout vertebrate genomes, including humans.

### Structural rearrangements and non-Mendelian inheritance

Two kinds of structural rearrangements in the genomes of wild mice have provided insight into genome evolution and into chromosome transmission during meiosis: Robertsonian (whole-arm) chromosomal translocations (also called fusions) and t-haplotypes, which are associated with chromosomal inversions (see ‘Glossary’). The karyotype of wild mice typically consists of 2n = 40 acrocentric chromosomes. However, numerous populations exist within the subspecies *M. m. domesticus* in which the diploid number is as low as 2n = 22 as a result of Robertsonian fusions between two different acrocentric chromosomes, creating a metacentric chromosome (e.g., Gropp et al., 1972; Capanna et al., 1973). Otherwise indistinguishable from mice with the standard karyotype, some karyotypic races show some reproductive isolation as a consequence of the mis-pairing of chromosomal heterozygotes in hybrids and the production of aneuploid gametes or complete meiotic arrest (reviewed in Garagna et al., 2014). Interestingly, some Robertsonian heterozygotes show transmission distortion in meiosis, preferentially transmitting the acrocentric products (instead of the fused, metacentric chromosome) to the oocytes in female meiosis (Gropp and Winking, 1981). In these cases, the metacentric chromosomes are preferentially distributed to the first polar body.

T-haplotypes are meiotic-drive systems (see ‘Glossary’) found at low frequency in natural populations (Ardlie and Silver, 1996). T-alleles are associated with inversions on chromosome 17, and they show a strong transmission distortion in meiosis, with heterozygotes transmitting the t-bearing allele to over 90% of gametes (Lyon, 2003). The responder locus (see ‘Glossary’) was first identified and molecularly characterized 15 years ago (Herrmann et al., 1999), but transmission distortion was first documented in 1927 (reviewed in Willison and Lyon, 2000; Herrmann and Bauer, 2012). Decades of research have made t-haplotypes one of the best-studied meiotic-drive systems in any organism, providing a model for understanding non-Mendelian inheritance.

### Adaptation: rodenticide resistance

One consequence of a commensal lifestyle is an ongoing arms race with humans intent on extirpating mice and reducing food loss and the spread of disease. Warfarin-based rodenticides were introduced in the 1950's and have been periodically replaced by other rodenticides that have anticoagulant properties owing to the development of resistance among rats and mice (e.g., reviewed in Pelz et al., 2005; Ishizuka et al., 2008; Buckle, 2011). The study of rodenticide resistance in wild populations has provided insights into the molecular biology and biochemistry of anticoagulants and mechanisms of resistance to those agents. Importantly, the spread of rodenticide resistance has also led to insights into the process of adaptation (e.g., Rost et al., 2004, 2009; Pelz et al., 2005). For example, Song et al. (2011) found that an allele of the mouse gene *Vkorc1*, which underlies resistance to warfarin in *M. m. domesticus*, resulted from adaptive introgressive hybridization (see ‘Glossary’) with the Algerian mouse (*M. spretus*). This study is one of many recent studies that highlight that adaptive introgression is an often overlooked source of new variation in animals (Hedrick, 2013).

### Speciation and hybrid male sterility

Laboratory crosses between some subspecies of house mice produce hybrid males that are sterile or have reduced fertility (e.g., Forejt and Ivanyi, 1974; Britton-Davidian et al., 2005). In nature, house mouse subspecies hybridize where they meet in secondary contact. The best-studied hybrid zone is between *M. m. domesticus* and *M. m. musculus* in Central Europe (e.g., Vanlerberghe et al., 1986; Dod and Jermlin, 1993, 2005; Payseur et al., 2004; Macholán et al., 2007; Teeter et al., 2008; Wang et al., 2011; Janoušek et al., 2012). This hybrid zone is young; colonization of this area is relatively recent (∼3000 YA; for example, Cucchi et al., 2005; reviewed in Baird and Macholán, 2012). Studies of this hybrid zone, and of laboratory crosses between these subspecies, have helped us to understand speciation genetics, particularly the genetic basis of hybrid male sterility (e.g., Mihola et al., 2009; White et al., 2011; Turner et al., 2014). For example, evidence from natural populations and from laboratory crosses has revealed an important role of the X chromosome in reproductive incompatibilities (Tucker et al., 1992; Oka et al., 2004; Storchová et al., 2004; Dod et al., 2005; Good et al., 2008; Oka and Shiroishi, 2012), a pattern that appears to be quite general across animals (Coyne and Orr, 2004). Studies of wild house mice have also found that hybrid male sterility has a complex basis, involving many genes (Oka et al., 2007; Good et al., 2008; White et al., 2011, 2012; Turner and Harr, 2014). Laboratory crosses between *M. m. domesticus* and *M. m. musculus* also led to the positional cloning of *Prdm9*, the only gene known to contribute to hybrid sterility in vertebrates (Mihola et al., 2009). This gene has a role in recombination rate variation in both mice and humans (e.g., Baudat et al., 2010; Berg et al., 2010; Parvanov et al., 2010). The identification of other genes underlying hybrid male sterility remains a challenge, but the combination of mapping studies in the lab and studies of regions showing limited introgression in nature might identify good candidates for future study (Janoušek et al., 2012; Phifer-Rixey et al., 2014; Turner and Harr, 2014).

## Conclusions: the next frontiers

As outlined in Box 3, much remains to be learned about wild mice, even in the areas of research highlighted above. Genetic variation, phenotypic variation, variation in disease and parasite load remain uncharacterized in many wild populations throughout the world. The genetic bases for adaptive phenotypes are largely unknown. Contact zones between subspecies outside of Europe are mostly unexplored. Wild mice are an untapped reservoir of genetic variation. The generation of new wild-derived inbred strains would add a valuable component to existing mouse resources by adding new genetic variants. Moreover, since haplotype blocks are much shorter in wild mice than in classical inbred strains (Laurie et al., 2007), association mapping (see ‘Glossary’) with wild mice could help link individual genes to phenotypic differences. New more efficient genome-editing methods (Jinek et al., 2012) will also enable the direct testing of the effects of wild alleles on phenotypes. Thus, there is great potential for wild mice to continue to fuel new advances in the biosciences.

10.7554/eLife.05959.005

Box 3. **Outstanding questions about the natural history of house mice.**

Although house mice have been studied for more than a century, there are still important questions about the basic biology of wild house mice that remain largely unanswered. Answers to these questions would further strengthen the mouse as a model for research.

- What is the nature and extent of variation in morphology, physiology, reproduction, and development among wild house mice that have adapted to live in different environments? Although house mice are known to occur in a wide variety of environments, we still know relatively little about their physiological ecology. For example, how do some house mice survive extreme cold, high elevations, or extremely arid regions? Further study of such populations would likely provide additional mouse models for important phenotypes.
- What are the determinants of social structure in mice? Mice sometimes live in small demes and sometimes live in larger aggregations, but much remains to be learned about the causes of these differences.
- Which pathogens and parasites are present in house mice from different areas? Infectious agents can be a powerful evolutionary force, yet we know little about natural infections of mice from different places. Pathogens are likely to vary between temperate and tropical areas, but this remains largely uninvestigated. Similarly, mice from different areas may have evolved resistance to different pathogens, but this too is mostly unstudied.
- What determines the limits to the distribution of house mice? They are amazingly successful at colonizing new areas, but they are not found everywhere. How important is competition with native rodents in determining the distribution of house mice?
- Which genes underlie adaptation? Our understanding of the genetic basis of adaptive differences is in its infancy. There have been some genome scans for selection, but few instances in which specific genes have been linked to specific phenotypes.
- What is the structure of haplotype blocks in natural wild mouse populations? Understanding haplotype structure is important for characterizing and understanding the evolution of recombination and will also lay the groundwork for association studies using wild mice.

**DOI:** http://dx.doi.org/10.7554/eLife.05959.005
